# A comparison of the main outcomes from BP-BES and DP-DES at five years of follow-up: A systematic review and meta-analysis

**DOI:** 10.1038/s41598-017-14247-6

**Published:** 2017-11-03

**Authors:** Pan Lu, Shuai Lu, Yuanyuan Li, Mengmeng Deng, Zhaohui Wang, Xiaobo Mao

**Affiliations:** 1 Department of Cardiology, Union Hospital, Tongji Medical College, Huazhong University of Science and Technology, Wuhan, 430022 China; 2 Department of Cardiology, Tongji Hospital, Tongji Medical College, Huazhong University of Science and Technology, Wuhan, 430030 China

## Abstract

Biodegradable polymer biolimus-eluting stents (BP-BES) are third-generation drug-eluting stents (DES) composed of biodegradable polymers that may improve prognosis after percutaneous coronary intervention (PCI). After five years of follow-up, BP-BES showed conflicting results compared to durable polymer drug-eluting stents (DP-DES). We performed a meta-analysis of the outcomes of studies on BP-BES and DP-DES after percutaneous coronary intervention (PCI) at five years of follow-up. Eligible studies were retrieved from PubMed, Embase and the Cochrane Library and reported the results of all-cause mortality, myocardial infarction (MI), target lesion revascularization (TLR), target vessel revascularization (TVR) and stent thrombosis (ST) at five years of follow-up. Five studies of a total of 4687 patients were included in the meta-analysis. At five years of follow-up, BP-BES was associated with lower rates of major adverse cardiac events (MACE) (OR = 0.83, 95%CI = [0.71, 0.97]), TLR (OR = 0.77, 95%CI = [0.62, 0.96]) and ST (OR = 0.60, 95%CI = [0.43 to 0.84]), whereas no significant differences in mortality, MI, or TVR rates were detected. Our results demonstrated that at five years of follow-up, BP-BES can significantly reduce the risk of MACE, TLR and ST, which indicate that safety and efficacy were increased after PCI.

## Introduction

Application of drug-eluting stents (DES) has had a great impact on percutaneous coronary intervention (PCI). Compared with bare metal stents (BMS), a reduced risk of restenosis and target lesion revascularization were observed with DES in previous clinical trials^1–4^. Because of their efficacy in limiting neointimal hyperplasia, DES were treated as a standard therapy in PCI. Self-perpetuating inflammation and late stent thrombosis (ST) were associated with the durable polymer used in the first- and second-generation DES^5–11^. To overcome these adverse events, biodegradable polymer drug eluting stents (BP-DES) were developed.

Biolimus is a highly lipophilic sirolimus analogue that inhibits the proliferation of smooth muscle cells by binding to the FK-binding protein and subsequently inhibiting mammalian target of rapamycin (mTOR)^12^. Biolimus-eluting stents (BES) are third-generation DES and elute biolimus from a polylactic acid (PLA) biodegradable polymer applied to the stent’s abluminal surface^13^. BES include Nobori stents (Terumo, Tokyo, Japan) and Biomatrix stents (Biosensors Europe SA, Morges, Switzerland). After implantation, the biodegradable polymer gradually dissolves into water and carbon dioxide within nine months^14,15^, and alleviates self-perpetuating inflammation and late stent thrombosis. Therefore, biodegradable polymer biolimus-eluting stents (BP-BES) may play an important role in reducing the risk of persistent inflammation and ST.

Some studies have compared BP-BES and durable polymer drug-eluting stents (DP-DES) in terms of prognosis at five years of follow-up after PCI. However, the results were conflicting. BP-BES had an advantage in improved MACE as reported by Zhang *et al*.^16^. However, Chevalier *et al*.^17^ reported opposite results. The aim of this study was to perform a meta-analysis of the outcomes associated with BP-BES and DP-DES for the treatment of PCI at five years of follow-up.

## Results

### Characteristics of the Included Studies

**Figure 1:**
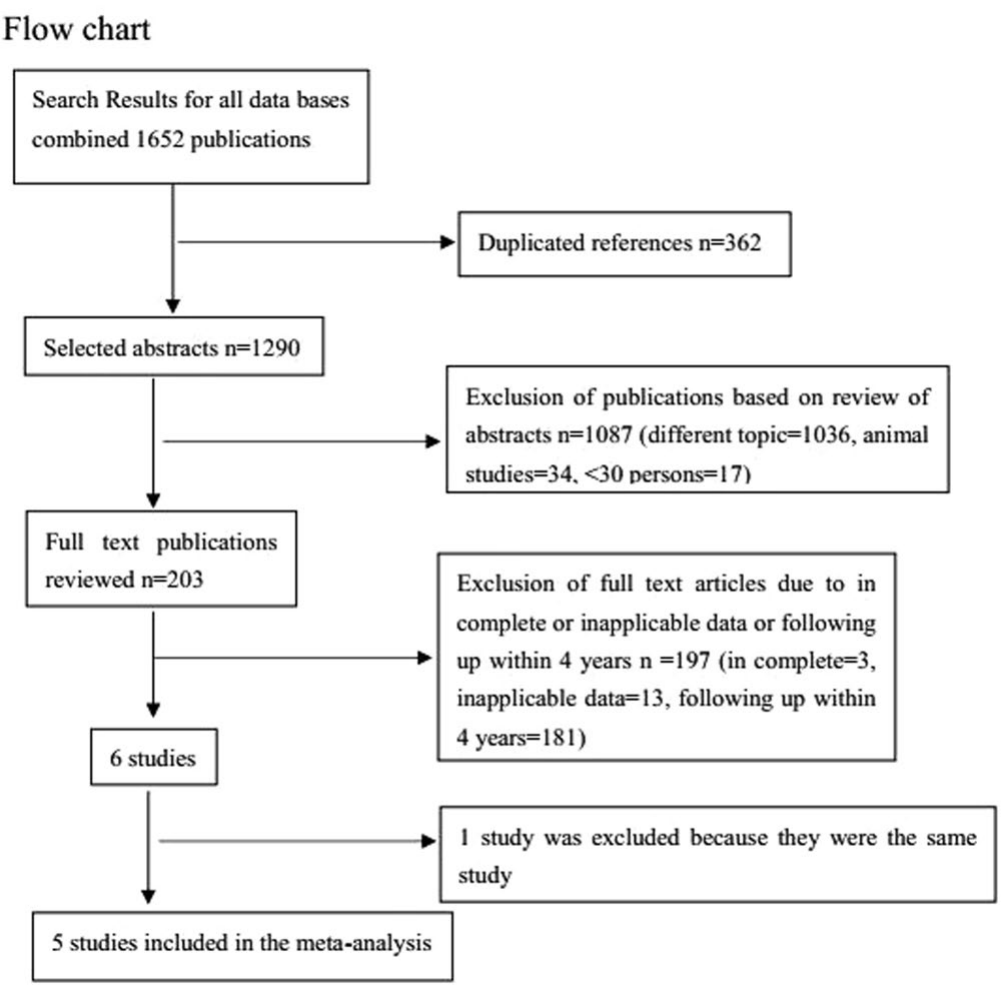
Flow chart of this meta-analysis that compared the main outcomes between BP-BES and DP-DES at five years of follow-up.

One-thousand-six hundred-fifty-two articles were obtained by online and manual searches. After removing duplicates and screening titles and abstracts, six independent trials that contained data for BP-BES versus DP-DES were included. The LEADERS (Limus Eluted From A Durable Versus ERodable Stent Coating) trial had four and five years of follow-up, and we obtained the latest data^13,18^. Finally, five studies^13,16,17,19,20^ were selected that included 4687 patients (2002 randomized to BP-BES and 2685 to DP-DES). (Fig. 1) (as seen in the flow chart).

The characteristics of the trials and patients are shown in Table 1. One trial^17^ compared BP-BES with paclitaxel-eluting stents (PES, Taxus Liberté/Express, Boston Scientific, Natick, MA, USA), three trials^13,16,19^ compared BP-BES with sirolimus-eluting stents (SES, Cypher Select, Cordis Corporation, Bridgewater, New Jersey, USA), and the remaining trial^20^ compared BP-BES with everolimus-eluting stents (EES, XIENCE V, Abbott Vascular, Santa Clara, CA; PROMUS, Boston Scientific, Inc., Natick, MA) or zotarolimus-eluting stents (ZES, RESOLUTE Integrity, Medtronic Vascular, Minneapolis, MN).

**Table 1:** Primary characteristics of the trials included in the study.

	Serruys 2013	Chevalier 2015	Zhang 2015	Grundeken 2016	Jaguszewski 2016
	BP-BES/DP-DES	BP-BES/DP-DES	BP-BES/DP-DES	BP-BES/DP-DES	BP-BES/DP-DES
Journal	JACC Cardiovasc Interv	Euro Intervention	Heart	Catheter Cardiovasc Interv	Catheter Cardiovasc Interv
BP-BES	BES BioMatrix Flex	BES Nobori	BES Biomatrix Flex	BES Biomatrix Flex	BES Biomatrix/Nobori
DP-DES	SES Cypher Select	PES Taxus Liberte/Express	SES Cypher Select	SES Cypher Select	EES Xience V/ZES Resolute Integrity
Sample size	857/850	238/125	280/293	258/239	369/1178
Age, years	64.6 ± 10.8/64.5 ± 10.7	63.2 ± 10.6/62.9 ± 10.0	62.9 ± 11.7/62.8 ± 11.7	65.1 ± 10.3/64.2 ± 11.0	62.3 ± 0.7/62.8 ± 0.3
Male, %	75.0/74.6	72.7/68.0	76.8/71.7	70.9/74.5	78.6/79.4
Diabetes (%)	26.0/22.5	16.8/27.2	50.5/59.2	65.4/57	15.7/18.1
ACS (%)	54.8/55.7	24.37/26.4	100/100	52.3/55.6	100/100
LVEF (%)	55.9 ± 11.3/55.4 ± 12.4	N/A	51.5 ± 10.1/51.4 ± 11.8	N/A	52.4 ± 0.7/52.7 ± 0.4
Multivessel disease (%)	24.2/20.7	N/A	N/A	N/A	54.2/56.2
SYNTAX score	13.2/13.3	N/A	14.7 ± 8.8/15.3 ± 8.7	16.9 ± 8.3/16.8 ± 8.9	N/A
No. of stents used per patient	1.3/1.3	N/A	2.2/2.2	N/A	N/A
Total stent length, mm per patient	24.7/24.6	N/A	26.6/27.9	N/A	N/A

BP-BES = biodegradable polymer biolimus-eluting stents, DP-DES = durable polymer drug-eluting stents, ACS = acute coronary syndrome, LVEF = left ventricular ejection fraction, N/A = not applicable.

All of the trials included five years of clinical follow-up data. The incidences of clinical outcomes at five years of follow-up are shown in Table 2. Dual antiplatelet therapy was administered to all patients for at least six or twelve months after discharge. Aspirin and clopidogrel were used in the majority of patients^13,16,17,19^; however, prasugrel or ticagrelor were also used^20^. Diabetes prevalence among the included trials ranged from 15.7–65.4%. The number of PCI patients with acute coronary syndrome ranged from 6.7%–100.0%.

**Table 2:** Clinical outcomes at five years of follow-up.

	MACE (BP-BES:DP-DES)	Death (BP-BES:DP-DES)	Cardiac death (BP-BES:DP-DES)	MI (BP-BES:DP-DES)	TVR (BP-BES:DP-DES)	TLR (BP-BES:DP-DES)	Definite or Probable ST (BP-BES:DP-DES)
Serruys 2013	186/857:216/850	79/857:87/850*	66/857:69/850	82/857:84/850	104/857:124/850	88/857:111/850*	31/857:44/850
Chevalier 2015	65/238:34/125	20/238:8/125	12/238:3/125	14/238:11/125	15/238:10/125	15/238:20/125	0/238:4/125
Zhang 2015	51/280:76/293	33/280:43/293	23/280:32/293	21/280:31/293	39/280:47/293	29/280:36/293	15/280:25/293
Grundeken 2016	29/258:31/239	26/258:22/239	17/258:11/239	10/258:13/239	22/258:20/239	17/258:16/239	1/258:7/239
Jaguszewski 2016	46/369:152/1178	13/369:57/1178	N/A	20/369:62/1178	22/369:58/1178	10/369:24/1178	6/369:22/1178

*The data from five years of follow-up were unpublished, so we included the data from four years of follow-up BP-BES = biodegradable polymer biolimus-eluting stents, DP-DES = durable polymer drug-eluting stents, MACE = major adverse cardiac events, MI = myocardial infarction, PCI = percutaneous coronary intervention, TLR = target lesion revascularization, TVR = target vessel revascularization, ST = stent thrombosis, N/A = not applicable.

The pooled ORs and 95%CIs of the comparisons of the main outcomes between DP-BES and BP-DES are shown in Table 3. Three outcomes were significantly different: MACE, TLR and ST; the results of death, MI and TVR were not significantly different.

**Table 3:** Pooled ORs and 95% CIs of the main outcomes between DP-BES and BP-DES.

Main Outcomes	NO. of studies	OR	95%CI	Statistical method	*I* ^2^ (%)	P^a^_heterogeneity_
MACE	5	**0.83**	**[0.71, 0.97]**	Fixed	0	0.56
Death	5	0.89	[0.72, 1.11]	Fixed	0	0.70
MI	5	0.88	[0.70, 1.10]	Fixed	0	0.70
TVR	5	0.89	[0.73, 1.08]	Fixed	0	0.68
TLR	5	**0.77**	**[0.62, 0.96]**	Fixed	45	0.12
ST	5	**0.60**	**[0.43, 0.84]**	Fixed	27	0.24

OR = odd ratio; CI = confidence interval, BP-BES = biodegradable polymer biolimus-eluting stents, DP-DES = durable polymer drug-eluting stents, MACE = major adverse cardiac events, MI = myocardial infarction, TLR = target lesion revascularization, TVR = target vessel revascularization, ST = stent thrombosis. ^a^P value for between-study heterogeneity based on the Q test. Significant results are presented in bold.

### Clinical Endpoints

**Figure 2:**
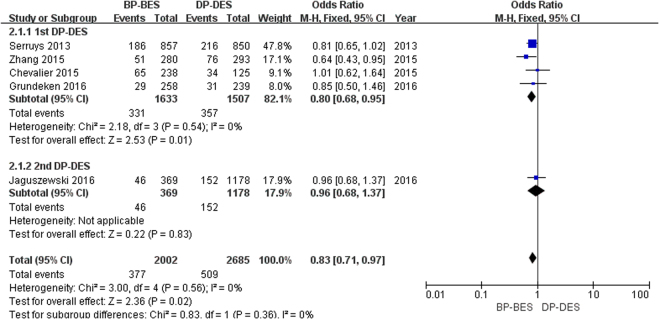
Forest plot of MACE (major adverse cardiac events) between BP-BES (biodegradable polymer biolimus-eluting stents) and DP-DES (durable polymer drug-eluting stents), OR = odd ration, CI = confidence interval.

As seen in Figure 2, a total of 886 patients (18.9%) had MACE. In the first-generation durable polymer drug-eluting stents (1st DP-DES) group, the use of BP-BES significantly reduced the risk of MACE compared to the- 1st DP-DES (20.3% versus 23.7%; OR [95% CI] = 0.80 [0.68, 0.95], P = 0.01). In the second-generation durable polymer drug-eluting stents (2nd DP-DES) group, there was no significant difference in the risk of MACE between BP-BES and 2nd DP-DES (12.5% versus 12.9%; OR [95% CI] = 0.96 [0.68, 1.37], P = 0.83). Finally, BP-BES significantly reduced the risk of MACE compared to DP-DES (18.8% versus 19.0%; OR [95% CI] = 0.83 [0.71, 0.97], P = 0.02; I^2^ = 0%, P_heterogeneity_ = 0.56). No heterogeneity was found across the included trials.

**Figure 3:**
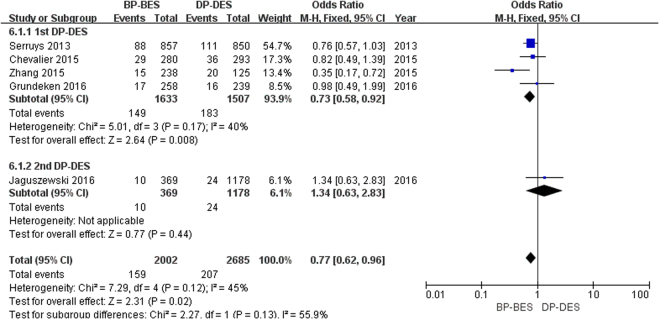
Forest plot of TLR (target lesion revascularization) between BP-BES (biodegradable polymer biolimus-eluting stents) and DP-DES (durable polymer drug-eluting stents), OR = odd ration, CI = confidence interval.

As seen in Figure 3, 366 patients (7.8%) underwent repeat revascularization of the target lesion. In the 1st DP-DES group, the use of BP-BES significantly reduced the risk of TLR compared to 1st DP-DES (9.1% versus 12.1%; OR [95% CI] = 0.73 [0.58, 0.92], P = 0.008). In the 2nd DP-DES group, there was no significant difference in the risk of TLR between BP-BES and 2nd DP-DES (2.7% versus 2.0%; OR [95% CI] = 1.34 [0.63, 2.83], P = 0.44). Finally, BP-BES was superior to DP-DES in reducing the risk of TLR (7.9% versus 7.7%; OR [95% CI] = 0.77 [0.62–0.96], P = 0.02; I^2^ = 45%, P_heterogeneity_ = 0.12). No heterogeneity was found across the included trials.

**Figure 4:**
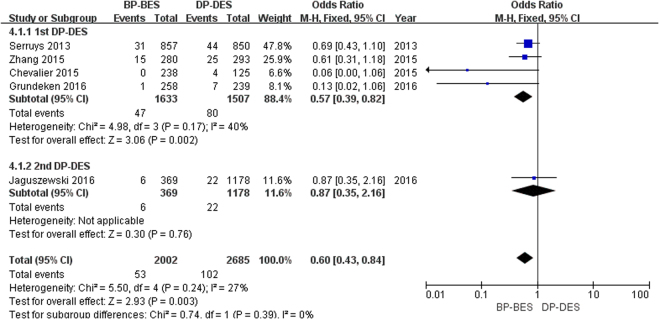
Forest plot of ST (stent thrombosis) between BP-BES (biodegradable polymer biolimus-eluting stents) and DP-DES (durable polymer drug-eluting stents), OR = odd ration, CI = confidence interval.

Definite/probable ST was observed in a total of 155 patients (3.3%), as presented in Fig. 4. In the 1st DP-DES group, the use of BP-BES significantly reduced the risk of ST compared to 1st DP-DES (2.9% versus 5.3%; OR [95% CI] = 0.57 [0.39, 0.82], P = 0.002). In the 2nd DP-DES group, there was no significant difference in the risk of ST between BP-BES and 2nd DP-DES (1.6% versus 1.9%; OR [95% CI] = 0.87 [0.35, 2.16], P = 0.76). Finally, BP-BES significantly reduced the risk of ST compared to DP-DES (2.6% versus 3.8%; OR [95% CI] = 0.60 [0.43–0.84], P = 0.003; I^2^ = 27%, P_heterogeneity_ = 0.24). No heterogeneity was found across the included trials.

**Figure 5:**
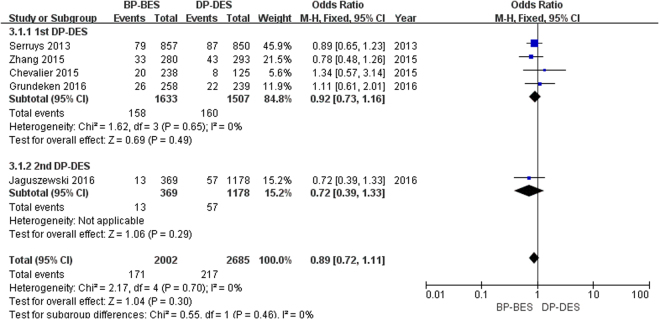
Forest plot of death between BP-BES (biodegradable polymer biolimus-eluting stents) and DP-DES (durable polymer drug-eluting stents), OR = odd ration, CI = confidence interval.

As shown in Fig. 5, a total of 388 patients (8.3%) died. BP-BES showed no superiority in reducing the risk of death compared to 1st DP-DES (9.7% versus 10.6%; OR [95% CI] = 0.92 [0.73, 1.16], P = 0.49) or the 2nd DP-DES (3.5% versus 4.8%; OR [95% CI] = 0.72 [0.39, 1.33], P = 0.29). There was no significant difference in the risk of death between BP-BES and DP-DES (8.5% versus 8.1%; OR [95% CI] = 0.89 [0.72, 1.11], P = 0.30; I^2^ = 0%, P_heterogeneity_ = 0.70).

**Figure 6:**
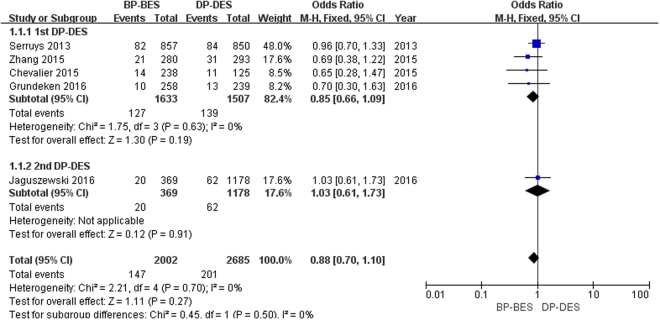
Forest plot of MI (myocardial infarction) between BP-BES (biodegradable polymer biolimus-eluting stents) and DP-DES (durable polymer drug-eluting stents), OR = odd ration, CI = confidence interval.

As shown in Fig. 6, a total of 348 patients (7.2%) had an MI. BP-BES showed no superiority in reducing the risk of MI compared to 1st DP-DES (7.8% versus 9.2%; OR [95% CI] = 0.85 [0.66, 1.09], P = 0.19) or 2nd DP-DES (5.4% versus 5.3%; OR [95% CI] = 1.03 [0.61, 1.73], P = 0.91). Finally, there was no significant difference in the risk of death between BP-BES or DP-DES (7.3% versus 7.5%; OR [95% CI] = 0.88 [0.70, 1.10], P = 0.27; I^2^ = 0%, P_heterogeneity_ = 0.70).

**Figure 7:**
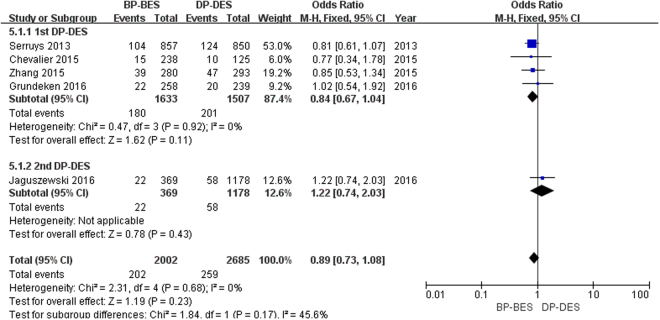
Forest plot of TVR (target vessel revascularization) between BP-BES (biodegradable polymer biolimus-eluting stents) and DP-DES (durable polymer drug-eluting stents), OR = odd ration, CI = confidence interval.

As shown in Fig. 7, a total of 461 patients (9.8%) required TVR. BP-BES showed no superiority in reducing the risk of MI compared to 1st DP-DES (11.0% versus 13.3%; OR [95% CI] = 0.84 [0.67, 1.04], P = 0.11) or 2nd DP-DES (6.0% versus 4.9%; OR [95% CI] = 1.22 [0.74, 2.03], P = 0.43). Finally, there was no significant difference in the risk of death between BP-BES and DP-DES (10.0% versus 9.6%; OR [95% CI] = 0.89 [0.73, 1.08], P = 0.23; I^2^ = 0%, P_heterogeneity_ = 0.68).

### Sensitivity analysis

**Figure 8:**
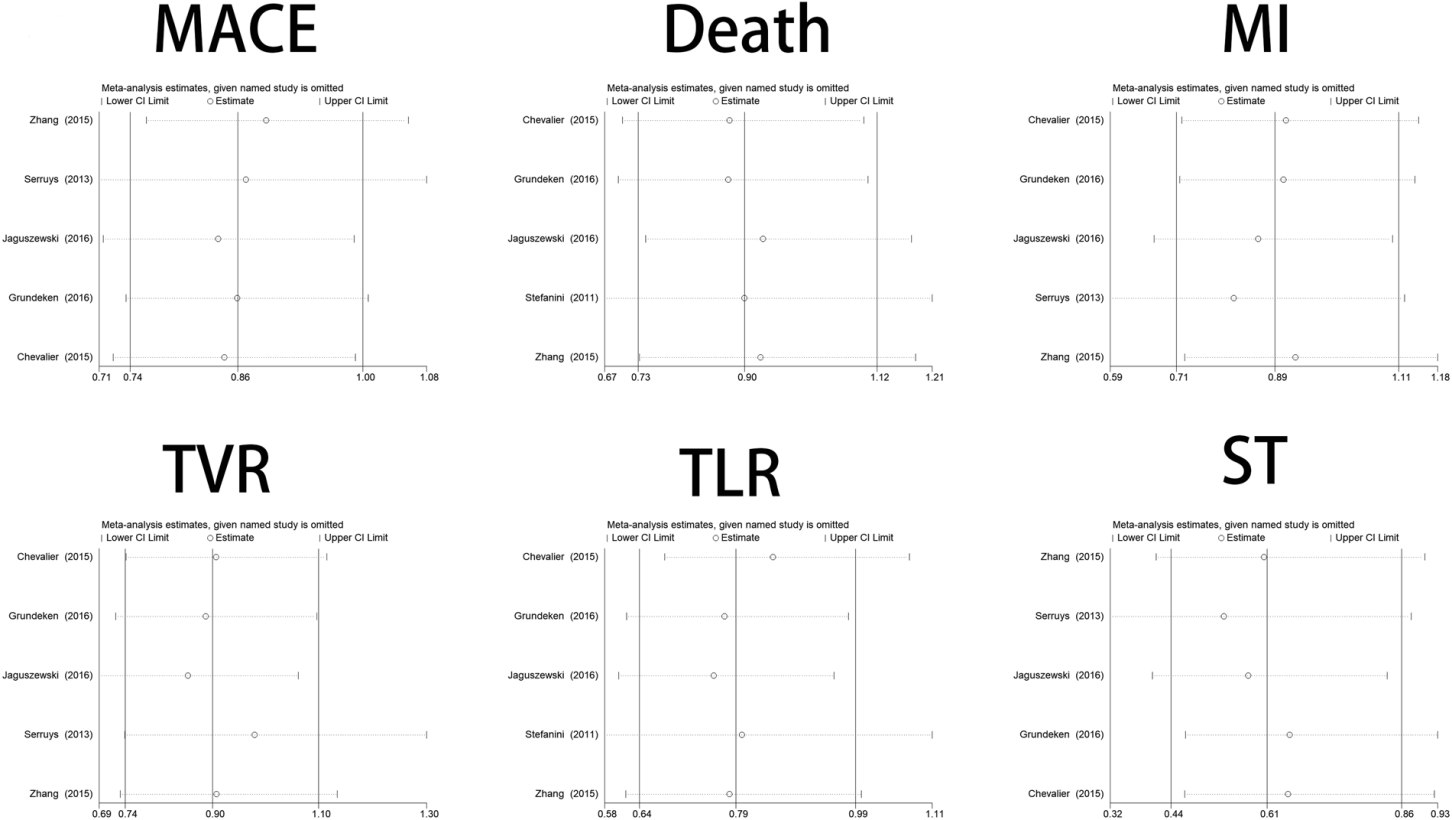
Influence analysis of the main outcomes between BP-BES (biodegradable polymer biolimus-eluting stents) and DP-DES (durable polymer drug-eluting stents).

A sensitivity analysis was performed by sequentially omitting 1 individual at a time to reflect the influence of each study on the overall meta-analysis. No heterogeneity was observed in the polymorphism (Fig. 8); thus, the results of our meta-analysis were stable.

## Discussion

Comparisons between BP-BES and DP-DES in terms of prognosis after PCI have attracted a great deal of attention. Many studies have been performed at different time points based on six outcomes (MACE, death, MI, TVR, TLR, ST), especially at one year^21–24^ or three years^25^ of follow-up. However, no significant evidence from one or three years of follow-up have supported the superiority of BP-BES or DP-DES. Our meta-analysis first compared the outcomes at five years of follow-up between BP-BES and DP-DES in terms of prognosis after PCI. The most inspiring finding of our meta-analysis was that BP-BES can significantly reduce the risk of MACE, TLR and ST without benefits on death, MI, or TVR.

MACE, as a composite endpoint of death; MI; and coronary revascularization were not unified in our study or in previous studies. In our study, at five years of follow-up, BP-BES demonstrated superiority in reducing the risk of MACE compared to 1st DP-DES (OR [95% CI] = 0.80 [0.68, 0.95], P = 0.01) and were not inferior to 2nd DP-DES (OR [95% CI] = 0.96 [0.68, 1.37], P = 0.83). Ultimately, BP-BES had a lower risk of MACE compared to DP-DES (OR 0.83, 95% CI = 0.71, 0.97), whereas the results of one and three years of follow-up demonstrated that there was no difference between BP-BES and DP-DES, as reported by Zhang *et al*. and Sakurai *et al*.

The difference between outcomes may attributed to the different time point of follow-up. The potential clinical advantage of the BP-BES might be expected to emerge once the biodegradable polymer has dissolved, and this may have occurred 9 months after implantation^14,15^. Thus, it was not surprising that BP-BES did not reduce the risk of MACE at one or even three years of follow-up. Moreover, only three trials were included at three years of follow-up; more trials with larger populations of patients are needed to support the conclusion. Five trials were included in our study; four^13,16,19,20^ of them demonstrated that BP-BES were associated with a lower risk of MACE than DP-DES. However, of these four trials, the results of three of the trials were not statistically significant (Serruys *et al*. OR [95% CI] = 0.81 [0.65, 1.02]; Jaguszewski *et al*. OR [95% CI] = 0.96 [0.68, 1.37]; Grundeken *et al*. OR [95% CI] = 0.85 [0.50, 1.46]). Finally, the results showed that BP-BES were superior in reducing the risk of MACE compared to DP-DES, and no heterogeneity was found across the included trials (I^2^ = 0%, P_heterogeneity_ = 0.56).

A lower risk of ST at a very long time (>one year, as defined by the Academic Research Consortium^26^) was observed in the BP-BES group^21,27^, which agreed with our study results. At five years of follow-up, BP-BES demonstrated superiority in reducing the risk of ST compared to 1st DP-DES (OR [95% CI] = 0.57 [0.39, 0.82], P = 0.002) and were not inferior to 2nd DP-DES (OR [95% CI] = 0.87 [0.35, 2.16], P = 0.76). Ultimately, BP-BES significantly reduced the risk of definite/probable ST (2.6% versus 3.8%; OR [95% CI] = 0.60 [0.43–0.84], P = 0.003). The results from each trial that was included in our study were not statistically significant (Serruys *et al*. OR [95% CI] = 0.69 [0.43, 1.10]^13^, Zhang *et al*. OR [95% CI] = 0.61 [0.31, 1.18]^16^, Chevalier *et al*. OR [95% CI] = 0.06 [0.00, 1.06]^17^, Grundeken *et al*. OR [95% CI] = 0.13 [0.02, 1.06]^19^, Jaguszewski *et al*. OR [95% CI] = 0.87 [0.35, 2.16]^20^), but all of them showed that BP-BES were associated with a lower risk of ST than DP-DES, so we archived an inspiring result. Incomplete endothelialization and the inflammatory response caused by the persistence of a durable polymer play important roles in very late ST^6,10^. Drug-eluting stents gradually release drugs from polymer coatings that are applied to the stent surface, which prolongs the time of completely endothelialization. Three to four months are required for complete endothelialization with BMS^28,29^, whereas with DES, more time is required^6^. At the same time, the level of endothelial coverage in BP- BES was comparable to that of BMS at four weeks, with no significant increase in inflammatory reactions up to 15 months^30^. Moreover, compared with DP-DES, BP-BES contains a biodegradable polymer that gradually dissolves into water and carbon dioxide, which are associated with a lower risk of inflammatory responses in animal studies^31^. Thus, these observations may explain why the BP-BES may be associated with a lower risk of very late ST and a better long-term outcome in our meta-analysis.

In our study, BP-BES demonstrated superiority in reducing the risk of TLR compared to 1st DP-DES (OR [95% CI] = 0.73 [0.58, 0.92], P = 0.008) and were not inferior to 2nd DP-DES (OR [95% CI] = 1.34 [0.63, 2.83], P = 0.44). Ultimately, BP-BES had a lower risk of TLR than DP-DES (OR [95% CI] = 0.77 [0.62–0.96]). However, a previous meta-analyses showed no difference between BP-BES and DP-DES at one and three years of follow-up^22,25,27,32^. The major reasons for this difference were as follows: first, the data in our included studies was collected at five years, which was longer than the above studies. Second, PES represented a weak competitor in comparison with SES and EES^33–35^. The number of PES in our meta-analysis was 2.7% (125/4687), which is higher than in the above studies (1.4% [125/9114]^22^, 1.0% [125/12090]^32^, and 0% [0/8436]^25^), which may contribute to the better result.

Based on our study we found no differences in mortality, MI, or TVR between BP-BES and DP-DES, but the insufficient sample size may contribute to this discrepancy. However, based on the current studies, we found that BP-BES could reduce the risk of MACE, ST and TLR compared to DP-DES. These results had statistical significance and no heterogeneity was found. Many elements may have resulted in this discrepancy, including the lack of an adequate sample size, differences between stents, experience of operators, presence of complications after PCI, seriousness of lesion, and so on.

BP-BES also had some disadvantages. In this study, after implantation of the BP-BES, 18.8% of patients presented with MACE, 7.9% of patients underwent TLR and 2.6% of patients had ST, which compromised the prognosis after PCI. Furthermore, after implantation, the biodegradable polymer gradually dissolved into water and carbon dioxide, alleviating self-perpetuating inflammation and late stent thrombosis, which in turn necessitates prolonged dual antiplatelet therapy that increases the risk of long-term bleeding events after PCI^36^.

There were several limitations in this meta-analysis. First, only English language articles were included in our study, which may bias the results. Second, patient heterogeneity and confounding factors might have affected the analysis. Third, significant heterogeneity was detected in some pooled analyses, which may have affected the meta-analysis results, even though we adopted the random effects model or introduced sensitivity analysis. Fourth, the number of included studies was relatively small, and the results should be interpreted with caution; further studies are needed to confirm these results.

In conclusion, BP-BES can significantly reduce the risk of MACE, TLR and ST compared with DP-DES at five years of follow-up, which indicates that BP-BES are associated with a better safety and efficiency after PCI in the long term.

## Materials and Methods

### Identification of Studies

PubMed, the Cochrane Library and Embase databases were thoroughly searched in September 2016 by the first two investigators to identify potential studies of BP-BES and DP-DES. The terms “Biolimus”, “Nobori”, “Biomatrix”, “BioFreedom”, and “stent” were used. Missing data (the data that we failed to identify during the electronic search) were obtained by reviewing the citations of review articles and all eligible studies.

### Inclusion and Exclusion Ceriteria

Citations were screened at the title and abstract level and retrieved as full reports. The inclusion criteria were: (1) comparison of BP-BES vs DP-DES; (2) studies reporting at least one of the following outcomes: MACE, all/cardiac death, MI, TVR, TLR, ST; and (3) clinical follow-up at five years. When more than one report of the same study was retrieved, the one with the longest follow-up was included. The exclusion criteria: (1) a duplication of previous publications; (2) a comment, review or editorial; and (3) a study without data. The studies were independently selected by two investigators, according to the inclusion and exclusion criteria by screening the title, abstract and full-text. Any dispute was resolved by discussion.

### Data Extraction

From each study, the following data were independently extracted by the first two investigators using a standardized form: first author’s last name, year of publication, journal, BP-BES, DP-DES, sample size, age, gender, patients with diabetes, patients with ACS, left ventricular ejection fraction (LVEF), multi-vessel disease, SYNTAS scores, numbers of stents used per patient, total stent length, and mm per patient. For data from multiple treatment groups, the approach recommended in the Cochrane handbook was adopted to avoid a unit- of –error analysis that may result from entering several comparisons into one meta-analysis, which could lead to “double-counts” of patients based on the same study. Disagreements were resolved through discussion.

### Statisticals analysis

RevMan version 5.3 (The Nordic Cochrane Centre, The Cochrane Collaboration, Copenhagen, Denmark) was used for statistical analyses. We calculated the odds risk (OR) and its 95%CI (confidence interval) for the five outcomes as binary data. Heterogeneity was evaluated by the magnitude of the Chi^2^, corresponding P value and I^2^ statistic. When the I^2^ value was above 50%, a random effects model based on the Mantel-Haenszel (MH) or inverse variance (IV) statistical approach was selected to combine the data. If the I^2^ value was below 50%, a fixed effects model based on the MH or IV statistical approach was selected, and a sensitivity analysis was conducted to detect the robustness of the result.
